# Nestin downregulation in rat vascular smooth muscle cells represents an early marker of vascular disease in experimental type I diabetes

**DOI:** 10.1186/s12933-014-0119-6

**Published:** 2014-08-21

**Authors:** Kim Tardif, Vanessa Hertig, Camille Dumais, Louis Villeneuve, Louis Perrault, Jean-François Tanguay, Angelino Calderone

**Affiliations:** Department of Biomedical Sciences, Université de Montréal, and Research Center, Montreal Heart Institute, Montreal, QC Canada; Departement of Physiology, Université de Montréal and Research Center, Montreal Heart Institute, Montreal, QC Canada; Research Center, Montreal Heart Institute, 5000 Belanger Street, Montreal, QC H1T 1C8 Canada; Department of Medicine, Université de Montréal and Research Center, Montreal Heart Institute, Montreal, QC Canada

**Keywords:** Type I diabetes, Rat, Vasculature, Nestin, Phosphohistone-3, Vascular smooth muscle cells, Hyperglycaemia

## Abstract

**Background:**

Nestin was reported to directly contribute to cell proliferation and the intermediate filament protein was detected in vascular smooth muscle cells. In experimental type I diabetes, nestin downregulation in the heart was identified as an incipient pathophysiological event. The following study tested the hypothesis that dysregulation of nestin expression in vascular smooth muscle cells represented an early event of vascular disease in experimental type I diabetes.

**Methods/Results:**

In the carotid artery and aorta of adult male Sprague-Dawley rats, a subpopulation of vascular smooth muscle cells co-expressed nestin and was actively involved in the cell cycle as reflected by the co-staining of nuclear phosphohistone-3. The infection of aortic vascular smooth muscle cells with a lentivirus containing a shRNAmir directed against nestin significantly reduced protein expression and concomitantly attenuated basal DNA synthesis. Two weeks following injection of adult male Sprague-Dawley rats with streptozotocin, the endothelial response of aortic rings to acetylcholine, vascular morphology and the total density of vascular smooth muscle cells in the vasculature of type I diabetic rats were similar to normal rats. By contrast, nestin protein levels and the density of nestin^(+)^/phosphohistone-3^(+)^-vascular smooth muscle cells were significantly reduced in type I diabetic rats. The *in vivo* observations were recapitulated *in vitro* as exposure of vascular smooth muscle cells to 30 mM D-glucose inhibited DNA synthesis and concomitantly reduced nestin protein expression.

**Conclusions:**

Hyperglycaemia-mediated nestin downregulation and the concomitant reduction of cycling vascular smooth muscle cells represent early markers of vascular disease in experimental type I diabetes.

**Electronic supplementary material:**

The online version of this article (doi:10.1186/s12933-014-0119-6) contains supplementary material, which is available to authorized users.

## Background

Type I diabetes accounts for 5-10% of the diabetic population and is characterized by the depletion of insulin synthesis secondary to pancreatic beta cell destruction [1]. Thus, hyper-glycaemia represents a chronic condition of type I diabetes and elevated plasma glucose levels was reported as the third major cause of global mortality [1]. Type II diabetes account for 90-95% of the diabetic population and is characterized by the reduced sensitivity of peripheral tissue to circulating insulin [1]. In this regard, a hypersecretion of insulin is chronically observed and ultimately leads to pancreatic beta cell exhaustion [1]. A major complication of diabetes is micro- and/or macrovascular disease in nearly all organs [1,2]. Microvascular complications include retinopathy, nephropathy, and neuropathy whereas macrovascular complications constitute the rapid acceleration of cardiovascular and cerebrovascular disease secondary to stroke [1,2]. An incipient pathophysiological event of diabetic vascular disease was an imbalance of homeostasis due to increased vasoconstriction secondary to impaired endothelial cell reactivity that occurred prior to the onset of overt clinical symptoms [1–3]. The underlying mechanisms included reduced bioavailability of nitric oxide secondary to the increased production of superoxide anion leading to the formation of peroxynitrite and/or compromised activity of endothelial nitric oxide synthase [1–3]. Vascular smooth muscle cell dysfunction likewise contributed to the progression of vessel disease in experimental models of diabetes and diabetic patients, albeit it remains presently unknown whether it occurred prior to, concomitantly or after impaired endothelial reactivity was established [1,2,4].

Nestin, a class VI intermediate filament protein was first detected in a population of neural progenitor/stem cells residing in the CNS [5]. However, several studies have identified nestin in developing skeletal myoblasts, endothelial cells during reparative angiogenesis and tumour vascularisation, upregulated in the infarcted heart, detected in diverse forms of cancer and a biological role in proliferation and/or migration was reported [6–13]. A recent study by Oikawa and colleagues demonstrated that nestin was expressed in vascular smooth muscle cells of the adult rat aorta [14]. Work from our lab detected a population of cardiac resident nestin-expressing cells that exhibited a neural progenitor/stem cell phenotype and downregulation of the intermediate filament protein was identified as an incipient pathophysiological event of type I diabetes [8,9,15,16]. Based on these observations, the present study tested the hypothesis that nestin expression in vascular smooth muscle cells (VSMCs) of the adult rat carotid artery and aorta was directly linked to proliferation and the intermediate filament protein was downregulated during the early stage of experimental type I diabetes attributed to hyperglycaemia.

## Methods

### Animal models

Vascular phenotype was determined in the aorta of neonatal Sprague-Dawleys rats (2-3 day old; Charles Rivers, Canada), carotid artery and aorta of adult male Sprague–Dawley rats (9–11 weeks old; Charles Rivers, Canada). Experimental type I diabetes was induced following a single injection of streptozotocin (60 mg/kg) in the jugular vein of adult male Sprague-Dawley rats (9–11 weeks old) [15,16]. Plasma glucose levels and left ventricular function were determined as previously described [16]. The use and care of laboratory rats was according to the Canadian Council for Animal Care and approved by the Animal Care Committee of the Montreal Heart Institute.

### Endothelial reactivity of aortic rings

The endothelial function of aortic rings was determined in organ chambers as previously described [17].

### Vessel morphology

Formalin fixed 6-8 μm thick sections of the carotid artery and aorta were stained with haematoxylin-phloxin-saffron (HPS) and images captured with the Olympus QICAM colour video camera interfaced with an Olympus CKX41 microscope. Vessel wall media thickness (mm) and media area (mm^2^) were measured with Image-Pro (version 7, Media Cybernetics, Rockville, MD).

### Vascular smooth muscle cells (VSMCs)

The carotid artery and aorta of adult male Sprague-Dawley rats (9–11 weeks old) were cut longitudinally and the lumen gently rubbed with a cotton swab to remove the endothelium. Vessel segments of 3-5 mm in length were digested in Dulbecco’s modified Eagle’s medium (DMEM; low glucose; HyClone Laboratories, Logan, UT) containing collagenase (type II; 1 mg/ml) for 5 hours at 37°C. Cells were filtered (40 μm nylon mesh; Corning, NY), cultured in DMEM supplemented with 10% FBS (Invitrogen Life Technologies, Grand Island, NY), 2% penicillin-streptomycin, 1% fungizone, epidermal growth factor (25 ng/ml), basic fibroblast growth factor (10 ng/ml) and grown until confluent. Experiments were subsequently performed on 1^st^/2^nd^ passage VSMCs plated at a density of 125-150 cells/mm^2^ in DMEM-containing 10% FBS for 24 hours. Thereafter, VSMCs were washed and the media replaced with DMEM supplemented with insulin/transferring/selenium (BD Bioscience, Bedford, MA) for 48 hours. To assess the effect of hyperglycaemia, VSMCs were plated in DMEM containing 5 mM D-glucose for 48 hours and thereafter supplemented with 25 mM D-glucose (Sigma, St-Louis MO), 30 mM L-glucose (Sigma) or 30 mM mannitol (Sigma) for 24 or 48 hours. DNA and protein synthesis was determined by ^3^H-thymidine and ^3^H-leucine uptake respectively, as previously described [10].

### Immunofluorescence

Formalin fixed 6-8 μm thick sections were subjected to the antigen retrieval method and stained with the mouse monoclonal anti-nestin (1:150; Chemicon, Temicula, CA), rabbit polyclonal anti-smooth muscle α-actin (1:100; Abcam, Cambridge, MA), goat monoclonal anti-CD31 (1:100; SantaCruz Biotechnologies, Santa Cruz, CA) or a rabbit polyclonal anti-phosphohistone-3 directed against phosphorylated serine 10 (1:100; Abcam). Primary and 1^st^/2^nd^ passage VSMCs were plated on glass coverslips for ~48 hours, fixed with 4% paraformaldehyde and stained with the mouse monoclonal anti-nestin (1:500; Chemicon), rabbit polyclonal anti-smooth muscle α-actin (1:200; Abcam), rabbit polyclonal anti-caldesmon (1:500; Abcam) or a rabbit polyclonal anti-smooth muscle-22α (1:1000; Abcam). The nucleus was identified with To-PRO-3 (InVitrogen; 1.5 μM) or 4′,6′-diamidino-2-phenylindole (DAPI, Sigma; 1.5 μM) and used to calculate total cell density normalized to the field (mm^2^). Secondary antibodies used were a goat anti-mouse IgG conjugated-Alexa-555 (1:800; InVitrogen) or a goat anti-rabbit IgG conjugated-Alexa-647 (1:800; InVitrogen). Immunofluorescence was visualized using a confocal LSM710 Zeiss microscope with the Zeiss LSM Image Browser. The density of nestin^(+)^- and phosphohistone-3^(+)^-VSMCs were determined with maximum projections derived from a z-stack (voxel size of 143x143x250 nm in XYZ) and normalized to the vessel area (mm^2^; average of at least 3-4 distinct fields). Non-specific staining was determined following the addition of the conjugated secondary antibody alone.

### Western blot

Lysates (30-50 μg) were prepared from the carotid artery, aorta or VSMCs, subjected to SDS-polyacrylamide gel (10%) electrophoresis and transferred to a PVDF membrane (Perkin Elmer Life Sciences, Boston, MA) [16]. Antibodies used include a mouse monoclonal anti-nestin (1:500; Chemicon), mouse monoclonal anti-eNOS (1:500; BD Bioscience), goat monoclonal anti-CD31 (1:500; SantaCruz Biotechnologies), rabbit polyclonal anti-caldesmon (1:2500; Abcam), rabbit polyclonal anti-smooth muscle-22α (1:5000; Abcam), rabbit polyclonal anti-smooth muscle α-actin (1:5000; Abcam), and mouse monoclonal anti-GAPDH (1:50,000; Ambion, Austin TX). Following overnight incubation at 4°C, the appropriate secondary antibody-conjugated to horseradish peroxidase (1:20,000, Jackson Immunoresearch, West Grove, PA) was added and bands visualized utilizing the ECL detection kit (Perkin Elmer). Films were scanned with Image J software® and the target protein signal was depicted as arbitrary light units normalized to GAPDH protein levels.

### Lentiviral construct

The lentiviral construct containing the shRNAmir directed against nestin was prepared as previously described [10]. The biological impact of the empty lentivirus and the lentivirus containing the shRNAmir directed against nestin was determined on DNA synthesis by measuring ^3^H-thymidine uptake of infected aortic-derived VSMCs, as previously described [10].

### Statistics

Data are presented as the mean ± S.E.M and (*n*) represents the number of rats or individual preparation of VSMCs used per experiment. Data was evaluated by a one-way ANOVA (GraphPad InStat) and a significant difference determined by the Student Newman-Keuls Multiple Comparisons post-hoc test or by a student’s unpaired t-test and a value of P < 0.05 considered statistically significant.

## Results

### Temporal and spatial pattern of nestin expression in the vasculature during physiological development

**Figure 1:**
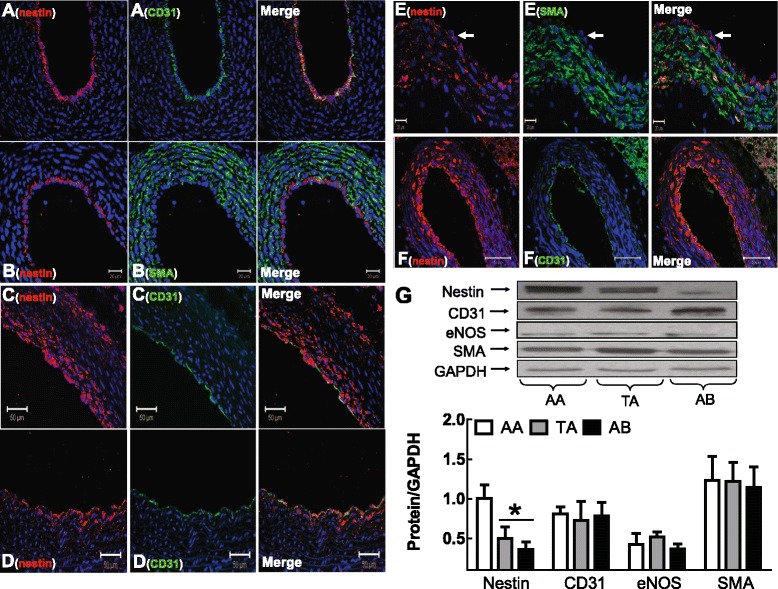
**Nestin expression in neonatal and adult rat vessels.** Nestin, CD31 and smooth muscle α-actin (SMA) staining of (Panels **A** & **B**) the neonatal aortic arch, (Panel **C**) adult aortic arch, (Panel **D**) adult thoracic region and (Panels **E** & **F**) adult rat carotid artery. The arrow indicates the luminal side and the nucleus identified by DAPI (blue fluorescence). (Panel **G**) Nestin expression was greatest in the aortic arch (AA) as compared to the thoracic (TA) and abdominal (AB) regions of the adult rat aorta, whereas CD31, eNOS and smooth muscle α-actin protein levels were unchanged. (*) Denotes P < 0.05 versus AA and protein levels were normalized to GAPDH.

In the aortic arch of 1-3 day old neonatal Sprague-Dawley rats, nestin immunoreactivity was detected in CD31^(+)^ endothelial cells (Figure 1A). Nestin staining of a subpopulation of smooth muscle α-actin^(+)^-VSMCs was variable among neonatal rats as expression was modest or completely absent in the media of the aortic arch (Figure 1B). In the aortic arch of adult male Sprague-Dawley rats, nestin immunoreactivity persisted in CD31^(+)^ endothelial cells and a subpopulation of VSMCs in the medial region expressed the intermediate filament protein (Figure 1C). Nestin immunoreactivity was identified in 20 ± 2% (N = 6, Figure 1C) of smooth muscle α-actin^(+)^-VSMCs residing predominantly in close proximity to the lumen of the adult aortic arch and occupied a density of 641 ± 89 cells/mm^2^ (N = 6). In the thoracic region of the adult rat aorta, a subpopulation of nestin^(+)^/smooth muscle α-actin^(+)^-VSMCs was also detected in the media but the percentage (11 ± 4%, Figure 1D) and density (276 ± 90 cells/mm^2^; N = 6) were significantly lower (P < 0.01) as compared to the aortic arch. The percentage (6 ± 2%) and density (170 ± 46 cells /mm^2^; N = 6) of nestin^(+)^/smooth muscle α-actin^(+)^-VSMCs in the abdominal aorta likewise remained significantly lower (P < 0.01) as compared to the aortic arch. The spatial disparity in the density of nestin^(+)^-VSMCs in the adult rat aorta was reaffirmed as expression of the intermediate filament protein was highest in the aortic arch and significantly lower in the thoracic and abdominal regions whereas smooth muscle α-actin protein levels were unchanged (Figure 1G). Nestin immunoreactivity of CD31^(+)^-endothelial cells persisted in the thoracic and abdominal regions and CD31 and eNOS protein expression was similar throughout the adult aorta (N = 3-4) (Figure 1G). In the carotid artery of adult male Sprague-Dawley rats, a subpopulation of smooth muscle α-actin^(+)^-VSMCs co-expressed nestin (26 ± 1%; 590 ± 72 cells/mm^2^;N = 4) and the intermediate filament protein was also detected in CD31^(+)^-endothelial cells (Figure 1E & F).

**Figure 2:**
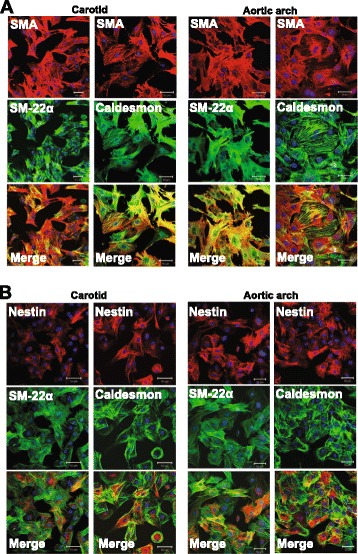
**Phenotype of cultured VSMCs.** (Panel **A**) Lineage specific markers were expressed in carotid artery and aortic VSMCs. (Panel **B**) Filamentous nestin was detected in caldesmon and smooth muscle-22α^(+)^-VSMCs. The nucleus was identified by To-PRO-3 (blue fluorescence).

To reaffirm nestin expression in a vascular smooth muscle cell lineage, carotid artery and aortic VSMCs were isolated, cultured and stained with smooth muscle α-actin, caldesmon and smooth muscle-22α. The vascular smooth muscle cell phenotype of primary and 1^st^/2^nd^ passage cells (N = 4-5) was confirmed and nestin-immunoreactive filaments were detected in caldesmon^(+)^/smooth muscle-22α^(+)^/smooth muscle α-actin^(+)^-VSMCs (Figure 2A & B).

### Biological role of nestin

**Figure 3:**
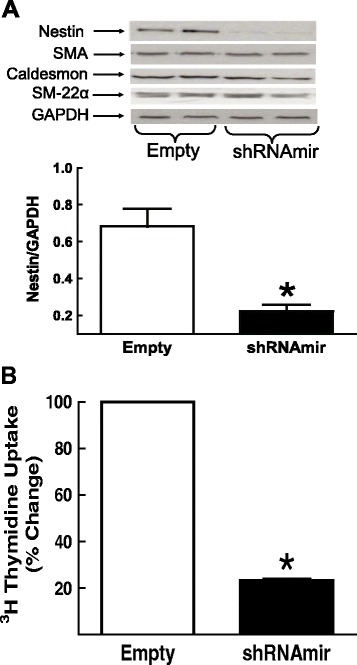
**Nestin depletion and DNA synthesis.** (Panel **A**) Aortic VSMCs infected with a lentivirus containing a shRNAmir directed against nestin reduced protein expression, whereas smooth muscle α-actin (SMA), caldesmon and smooth muscle-22α protein levels were unchanged. (Panel **B**) In nestin-depleted aortic VSMCs, ^3^H-thymidine uptake was significantly decreased. (*) Denotes P < 0.05 versus VSMCs infected with the empty lentivirus.

First passage aortic-derived VSMCs infected with a lentivirus containing a shRNAmir directed against nestin (N = 4) led to a significant reduction in the expression of the intermediate filament protein, whereas lineage specific markers were unaffected (Figure 3A). These data were reaffirmed by immunofluorescence as staining was diminished in aortic VSMCs infected with the lentivirus containing the shRNAmir directed against nestin (Additional file 1: Figure S1). DNA synthesis, as measured by ^3^H-thymdine uptake was significantly attenuated in nestin-depleted 1^st^ passage aortic VSMCs (N = 4) (Figure 3B).

**Figure 4:**
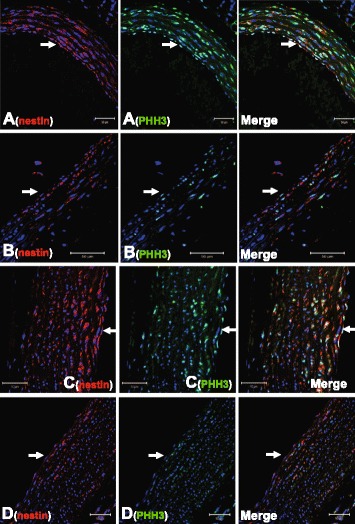
**Nestin** ^**(+)**^ **-VSMCs were actively involved in the cell cycle; impact of type I diabetes.** A subpopulation of nestin^(+)^-VSMCs and nestin^(+)^-endothelial cells in the (Panel **A**) carotid artery and (Panel **C**) aorta arch of normal adult rats were associated with nuclear PHH3 staining. In type I diabetic rats, the subpopulation of nestin^(+)^-VSMCs co-expressing nuclear PHH3 staining in the (Panel **B**) carotid artery and (Panel **D**) aortic arch was reduced and nestin/PHH3 co-staining of endothelial cells was also diminished. The arrow indicates the luminal side and the nucleus was identified by DAPI (blue fluorescence).

The established proliferative role of nestin suggested that a subpopulation of VSMCs expressing the intermediate filament protein in the vasculature of normal adult rats was actively engaged in the cell cycle. To evaluate cell cycle entry *in vivo*, phosphohistone-3 (PHH3) immunoreactivity characterized by the phosphorylation of serine 10 was examined [18]. Consistent with its proliferative role, a significant population of nestin^(+)^-VSMCs in the carotid artery and aortic arch of normal adult rats was associated with nuclear PHH3 staining (Figure 4A,C & Table 1). Furthermore, nestin^(+)^-endothelial cells in the vasculature of normal adult rats co-expressed nuclear PHH3 (Figure 4A & C).

**Table 1:** **Vascular remodeling of the carotid artery and aorta of normal and streptozotocin-induced type I diabetic rats**

	**Aortic arch**		**Carotid artery**	
	**Sham (N = 6)**	**STZ (N = 6)**	**Sham (N = 4-5)**	**STZ (N = 4-5)**
Total density (cells/mm^2^)	3114 ± 204	2693 ± 284	2869 ± 223	2710 ± 237
PHH3^(+)^ (cells/mm^2^)	1032 ± 201	137 ± 28*	797 ± 124	416 ± 108*
PHH3^(+)^/Nestin^(-)^ (cells/mm^2^)	645 ± 131	84 ± 18*	430 ± 73	273 ± 87*
Nestin^(+)^ (cells/mm^2^)	641 ± 89	385 ± 48*	590 ± 72	355 ± 64*
PHH3^(+)^/Nestin^(+)^ (cells/mm^2^)	387 ± 77	52 ± 15*	368 ± 62	143 ± 36*

STZ indicates streptozotocin-induced diabetic rats, data are presented as mean ± SEM, analyzed by unpaired t-test, (*) represents P < 0.05 versus sham and (N) number of rats examined.

### Streptozotocin-induced type I diabetes in the adult rat; endothelial reactivity and morphological/cellular vascular remodeling

**Figure 5:**
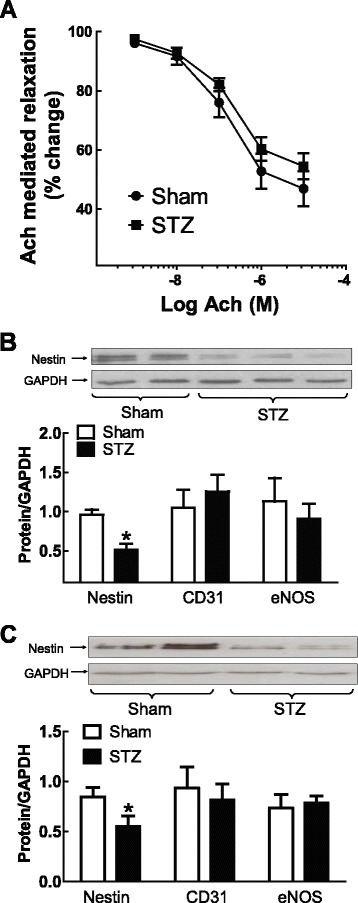
**Endothelial reactivity and nestin expression in type I diabetic rats.** (Panel **A**) Acetylcholine-mediated relaxation of pre-contracted aortic rings was comparable in sham (N = 3) and streptozotocin (STZ, N = 3) induced type I diabetic rats. Nestin expression in the (Panel **B**) carotid artery and (Panel **C**) aorta of type I diabetic rats was significantly reduced, whereas CD31 and eNOS levels were unchanged. (*) Denotes P < 0.05 versus sham and protein levels normalized to GAPDH.

Plasma glucose levels were increased and left ventricular contractility was significantly depressed 2 weeks after streptozotocin (STZ) injection of adult male rats (Table 2). Following pre-contraction with phenylephrine, acetylcholine treatment led to a dose-dependent relaxation of normal aortic rings and a quantitatively analogous response was observed in STZ-induced diabetic rats (Figure 5A). Consistent with the latter data, CD31 and eNOS protein levels (normalized to GAPDH) in the aorta and carotid artery of STZ-induced type I diabetic rats (N = 4-6) were comparable to normal rats (N = 4) (Figure 5B & C). Vascular morphology as measured by the media area and media thickness of the carotid artery and aorta were also similar in normal and type I diabetic rats (Table 3 & Additional file 2: Figure S2).

**Table 2:** **Cardiac function of normal and streptozotocin-induced type I diabetic rats**

	**BW**	**MAP**	**LVSP**	**LVEDP**	**LV + dP/dT**	**LV -dP/dT**	**Plasma glucose**
	**(g)**	**(mmHg)**	**(mmHg)**	**(mmHg)**	**(mmHg/sec)**	**(mmHg/sec)**	
Sham (N = 5)	312 ± 1	117 ± 3	148 ± 8	8 ± 1	6963 ± 188	6069 ± 172	10 ± 2
STZ (N = 5)	263 ± 2*	87 ± 1*	109 ± 1*	10 ± 1	5656 ± 79*	4155 ± 53*	29 ± 1*

STZ indicates streptozotocin-induced diabetic rats, BW, body weight, MAP, mean arterial pressure, LVSP, left ventricular systolic pressure, LVEDP, left ventricular end-diastolic pressure, +dp/dt, rate of contraction; -dp/dt rate of relaxation, LV, data are presented as mean ± SEM, analyzed by unpaired t-test, (*) represents P < 0.05 versus sham and (N) number of rats examined.

**Table 3:** **Vascular morphology of the carotid artery and aorta of normal and streptozotocin-induced type I diabetic rats**

	**Carotid artery**		**Aortic arch**		**Thoracic aorta**		**Abdominal aorta**	
	**Sham (N = 4)**	**STZ (N = 4)**	**Sham (N = 4)**	**STZ (N = 4)**	**Sham (N = 4)**	**STZ (N = 4)**	**Sham (N = 4)**	**STZ (N = 4)**
Medial thickness (mm)	0.063 ± 0.002	0.066 ± 0.002	0.15 ± 0.01	0.12 ± 0.01	0.11 ± 0.01	0.10 ± 0.01	0.10 ± 0.01	0.09 ± 0.01
Medial area (mm^2^)	0.147 ± 0.006	0.148 ± 0.011	0.82 ± 0.06	0.82 ± 0.04	0.59 ± 0.08	0.55 ± 0.05	0.44 ± 0.01	0.55 ± 0.01

STZ indicates streptozotocin-induced diabetic rats, data are presented as mean ± SEM, and (N) number of rats examined.

The total density of VSMCs (as measured by nuclear staining) in the carotid artery and aortic arch of STZ-induced type I diabetic rats and normal rats were not significantly different (Table 1). However, a significant loss in the density of VSMCs expressing nestin (Table 1) was observed in the carotid artery (Figure 4B) and aortic arch (Figure 4D) of type I diabetic rats. The data were reaffirmed as nestin protein expression was significantly reduced in the vasculature of type I diabetic rats as compared to normal rats (Figure 5B & C). The established proliferative role suggested that the reduced density of nestin^(+)^-VSMCs in the carotid artery and aortic arch of STZ-induced diabetic rats may be associated in part with a concomitant loss in the number of cycling cells. In the vasculature of STZ-induced diabetic rats, the density of PHH3^(+)^-VSMCs and nestin^(+)^-VSMCs co-expressing nuclear PHH3 were significantly decreased (Figure 4B & D; Table 1). Lastly, nestin and PHH3 staining was apparently reduced in endothelial cells lining the vasculature of type I diabetic rats (Figure 4B & D).

### Hyperglycaemia inhibited DNA synthesis and reduced nestin protein expression in VSMCs

**Figure 6:**
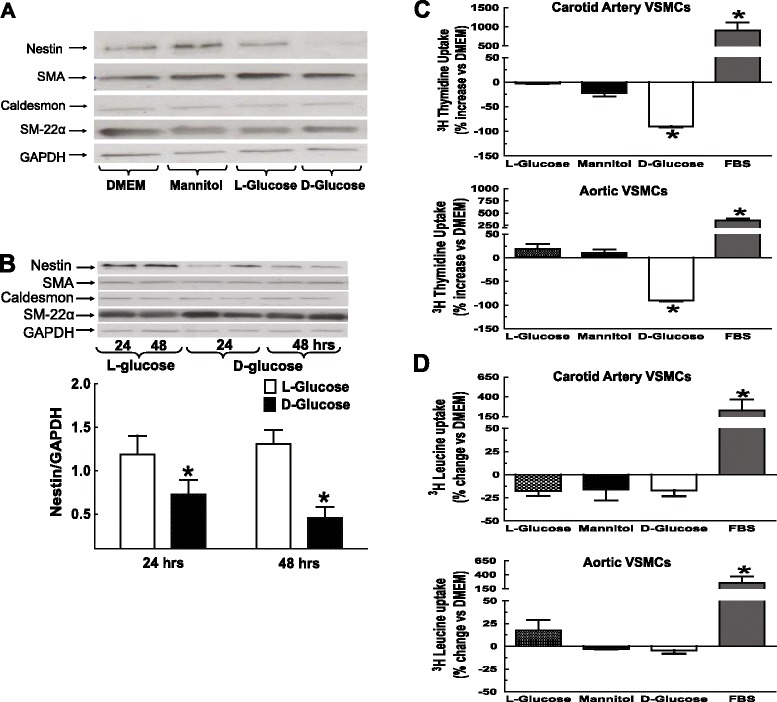
**Hyperglycaemia, nestin expression and DNA synthesis in VSMCs.** (Panels **A** & **B**) Exposure of 1^st^/2^nd^ passage carotid artery VSMCs to 30 mM mannitol or 30 mM L-glucose for 24 hours did not influence nestin protein expression as compared to DMEM. A 24 and/or 48-hour exposure to 30 mM D-glucose significantly reduced nestin protein levels, whereas smooth muscle α-actin (SMA), caldesmon and smooth muscle-22α protein expression was unchanged. (*) Denotes P < 0.05 versus L-glucose and protein levels normalized to GAPDH. (Panel **C**) VSMCs exposed to 30 mM D-glucose for 24 hours attenuated ^3^H-Thymidine uptake. VSMCs treated with 10% FBS increased ^3^H-thymidine uptake, whereas 30 mM mannitol and 30 mM L-glucose had no effect (Panel **D**) VSMCs exposed to 30 mM D-glucose, 30 mM L-glucose or 30 mM mannitol for 24 hours had no effect on ^3^H-Leucine uptake, whereas a robust increase was observed with 10% FBS. Results are presented as % change versus DMEM and (*) denotes P < 0.05 versus L-glucose/mannitol.

A 24 hour exposure of 1^st^/2^nd^ passage carotid artery and aortic VSMCs to 30 mM mannitol or L-glucose had no effect on nestin protein expression or lineage specific markers, as compared to cells treated with DMEM (N = 4-6) (Figure 6A & Additional file 3: Figure S3). The exposure of carotid artery VSMCs to 30 mM D-glucose for 24 and 48 hrs significantly downregulated nestin protein expression, whereas smooth muscle α-actin, caldesmon and smooth muscle-22α protein levels were unchanged (N = 4-6) (Figure 6A,B and Additional file 4: Figure S4). The exposure of aortic VSMCs to 30 mM D-glucose for 24 hrs did not have a significant effect on nestin protein expression whereas a downregulation of the intermediate filament protein was observed after a 48 hr exposure (N = 4-6) (Additional file 3: Figure S3). The 24 or 48 hr exposure of aortic VSMCs to 30 mM D-glucose did not influence the expression of lineage specific markers (Additional file 3: Figures S3 & Additional file 4: Figure S4). DNA synthesis, as measured by ^3^H-thymidine uptake was significantly decreased following a 24 hr exposure of VSMCs to 30 mM D-glucose (N = 4-6) (Figure 6C). By contrast, the density of VSMCs, as measured by nuclear staining (aortic VSMCs; L-glucose 51 ± 2 versus D-glucose 59 ± 7 cells/mm^2^; N = 3) and ^3^H-leucine uptake, a global index of protein synthesis were unchanged following exposure to 30 mM D-glucose (N = 4-6) (Figure 6D).

## Discussion

Nestin was first identified as a selective marker of CNS-derived neural progenitor/stem cells [5]. However, nestin expression was also reported in developing skeletal myoblasts, detected in endothelial cells during reparative angiogenesis and tumour vascularisation, upregulated in the infarcted heart and identified in diverse forms of cancer [6–11,19,20]. Oikawa and colleagues recently demonstrated that VSMCs of the adult rat aorta expressed nestin [14]. The present study has reaffirmed the latter findings and further revealed that nestin was also expressed in a subpopulation of VSMCs in the carotid artery of adult male rats. As reported by Oikawa and colleagues [14], a spatial disparity in the density of nestin^(+)^-VSMCs was identified as a greater population was observed in the aortic arch versus the thoracic and abdominal aortic regions of adult male rats. Consistent with the latter data, nestin protein expression was highest in the aortic arch and significantly lower in the thoracic and abdominal regions, whereas smooth muscle α-actin protein levels were comparable in the three regions of the aorta. Lastly, the *in vivo* immunofluorescence data was confirmed *in vitro* as nestin^(+)^-filaments were detected in primary and 1^st^/2^nd^ passage carotid artery and aortic VSMCs characterized by smooth muscle α-actin, caldesmon and smooth muscle-22α co-staining.

To ascertain the biological role, nestin expression was depleted with a lentivirus containing a shRNAmir that selectively targeted the intermediate filament protein [10]. Lentiviral shRNAmir-mediated depletion of nestin in aortic-derived VSMCs significantly attenuated basal ^3^H-thymidine uptake demonstrating that the intermediate filament protein participated in cell cycle entry. Based on these observations, an analogous paradigm may be prevalent in nestin-expressing VSMCs in the vasculature of adult rats. To examine the latter issue *in vivo*, immunoreactivity of phosphohistone H3 (PHH3); an established cell cycle protein that was directly phosphorylated on the residue serine 10 during chromosome condensation in G_2_/M phase was examined [18]. In the carotid artery and aortic arch of adult rats, a significant population of nestin^(+)^-VSMCs were actively engaged in the cell cycle as nuclear PHH3 co-staining was identified. Nestin staining of CD31^(+)^-endothelial cells in the vasculature of adult rats was also associated with nuclear PHH3 immunoreactivity. The latter findings were in stark contrast to that of Oikawa and colleagues [14] as their study did not detect nestin immunoreactivity in endothelial cells in the aorta of adult rats. Previous studies have reported nestin expression in proliferating endothelial cells in the developing pancreas, during wound healing and tumor vascularisation [8,11,19,20]. However, nestin^(+)^-endothelial cells in the carotid artery and aorta of normal adult male rats were also actively engaged in proliferation as revealed by nuclear PHH3 co-staining. Therefore, these data suggest that nestin expression in proliferating endothelial cells was not restricted to *de novo* blood vessel formation during physiological development and pathological remodeling.

Work from our lab has reported that nestin downregulation in the heart of type I diabetic rats was identified as an incipient pathophysiological event and contributed in part to the impaired neurogenic response of neural progenitor/stem cells during the reparative fibrotic response of the type I diabetic infarcted rat heart [15,16]. These observations provided the impetus to test the hypothesis that dysregulation of nestin expression in VSMCs may represent an early event of vascular disease in type I diabetes. Two weeks following streptozotocin (STZ) injection of adult male rats, plasma glucose levels were elevated and associated with left ventricular contractile dysfunction. Previous studies have reported that hyperglycemia-induced increase in oxidative stress was directly implicated in endothelial dysfunction and identified as an early pathological event of diabetes prior to the overt manifestation of symptoms [1–3]. In the present study, the vasorelaxant response of aortic rings to acetylcholine and eNOS and CD31 protein levels in the aorta during the early phase of experimental type I diabetes were similar to normal rats. In addition, the total density of VSMCs and morphological remodeling of the carotid artery and aorta were also comparable in normal and type I diabetic rats. However, nestin protein levels and the density of VSMCs expressing nestin and nuclear PHH3 were significantly reduced in the carotid artery and aortic arch of type I diabetic rats. These findings support the premise that despite the absence of a change in the total density of VSMCs in the vasculature of type I diabetic rats, a significant population was unable to re-enter the cell cycle as reflected by the concomitant downregulation of nestin and PHH3 expression. The immunofluorescence data further revealed that nestin and PHH3 staining was decreased in endothelial cells in the vasculature of type I diabetic rats, thereby suggesting that the proliferative response was also compromised.

*In vitro* experiments were performed to assess whether the reduced density of cycling VSMCs in STZ-induced diabetic rats was directly attributed to elevated plasma glucose levels. Previous studies have reported that cultured VSMCs exposed to high glucose significantly increased, decreased or had no effect on proliferation [21–24]. The underlying reasons for the disparate *in vitro* findings remain unknown. Therefore, to limit the potential spurious effects of long-term culturing, the impact of elevated glucose was examined exclusively on 1^st^/2^nd^ passage VSMCs. The 24 hour exposure of 1^st^/2^nd^ passage carotid artery and aortic VSMCs to 30 mM D-glucose significantly attenuated DNA synthesis, as reflected by the decreased uptake of ^3^H-thymidine. By contrast, the density of VSMCs and ^3^H-leucine uptake were unchanged following a 24 hour exposure to 30 mM D-glucose. The *in vitro* data recapitulated the *in vivo* findings as the total density of vascular smooth muscle cells was unchanged in the vasculature during the early phase of STZ-induced diabetes albeit a significant population was unable to re-enter the cell cycle. Moreover, the downregulation of nestin protein levels in VSMCs of STZ-induced diabetic rats was attributed in part to hyperglycemia as exposure of 1^st^/2^nd^ passage carotid artery and aortic VSMCs to 30 mM D-glucose significantly reduced expression, whereas lineage specific markers were unaffected. A similar paradigm was reported following renal damage secondary to experimental type I diabetes as nestin expression was reduced in podocytes mediated by elevated plasma glucose levels [25,26]. Thus, the hyperglycemic environment of experimental type I diabetes contributed in part to the loss of nestin expression in the vasculature and downregulation of the intermediate filament protein may further represent an incipient event attenuating the re-entry of VSMCs in the cell cycle.

A seminal finding of the present study was the apparent physiological turnover of a subpopulation of nestin^(+)^-VSMCs in the carotid artery and aorta of normal adult male rats characterized by concomitant nuclear PHH3 staining. During the early phase of experimental type I diabetes, endothelial reactivity, vessel morphology and the total density of VSMCs were similar to normal rats. However, the density of VSMCs co-expressing nestin and PHH3 in the vasculature of STZ-induced diabetic rats was significantly reduced. The latter paradigm was recapitulated *in vitro* as the acute exposure of VSMCs to 30 mM D-glucose significantly reduced nestin protein levels and attenuated DNA synthesis. Collectively, these data support the novel premise that hyperglycaemia-mediated nestin downregulation and the concomitant reduction of cycling VSMCs represent early markers of vascular disease in experimental type I diabetes that occurred prior to the onset of impaired endothelial reactivity.

## Additional files

Additional file 1: Figure S1. Lentivirus-sHRNAmir directed against nestin. (Panel **A**) The infection of aortic vascular smooth muscle cells with the empty lentivirus expressing the reporter green fluorescent protein (GFP) had no significant effect on nestin immunoreactivity. (Panel **B**) The infection of aortic vascular smooth muscle cells with a lentivirus containing a shRNAmir directed against nestin significantly reduced staining of the intermediate filament protein. Additional file 2: Figure S2. Vascular morphology. The media thickness and area of the carotid artery (CA) and aortic arch (AA) of streptozotocin (STZ) induced type I diabetic rats were similar to sham rats. Additional file 3: Figure S3. Hyperglycaemia downregulated nestin protein expression in aortic VSMCs. The exposure of (Panels **A** & **B**) aortic VSMCs to 30 mM mannitol or 30 mM L-glucose for 24 hrs did not significantly influence nestin protein expression as compared to DMEM. A 48 hr exposure of aortic-derived VSMCs to 30 mM D-glucose significantly reduced nestin protein levels, as compared to 30 mM L-glucose treated cells, whereas smooth muscle α-actin (SMA), caldesmon and smooth muscle-22α protein expression was unchanged. (*) Denotes *p*< 0.05 versus L-glucose and data normalized to GAPDH. Additional file 4: Figure S4. The impact of hyperglycaemia on vascular smooth muscle cells. A 24 and/or 48 hour exposure of (Panel **A**) carotid artery (n=4-6) and (Panel **B**) aortic derived (n=4-6) vascular smooth muscle cells to 30 mM D-glucose had no significant effect on smooth muscle α-actin, caldesmon and smooth muscle-22α protein expression as compared to 30 mM L-glucose. Proteins levels were normalized to GAPDH.
