# Supplementary figures and images for: Nestin downregulation in rat vascular smooth muscle cells represents an early marker of vascular disease in experimental type I diabetes
Source: Cardiovasc Diabetol. 2014 Aug 21;13:119. doi: 10.1186/s12933-014-0119-6 (PMC4143548; doi:10.1186/s12933-014-0119-6)

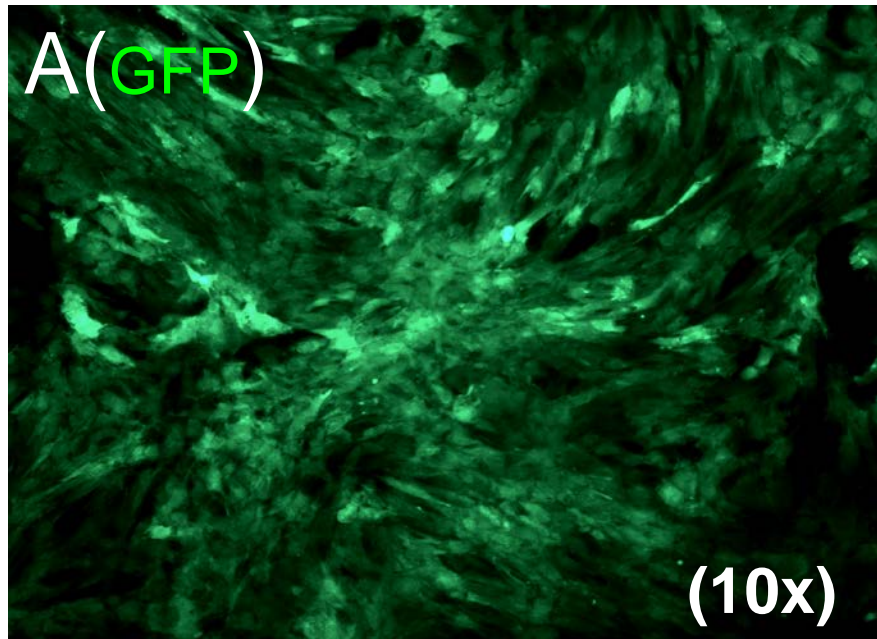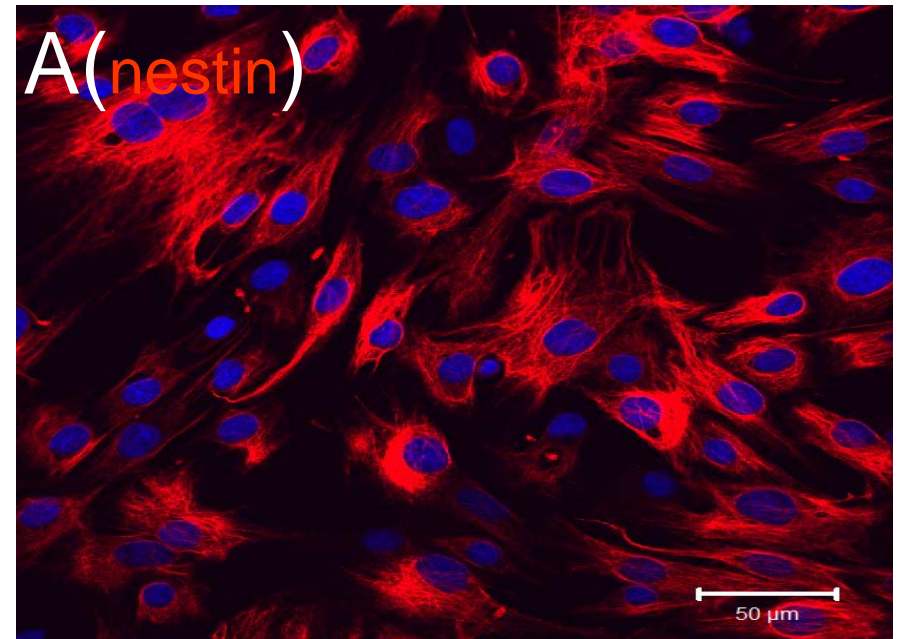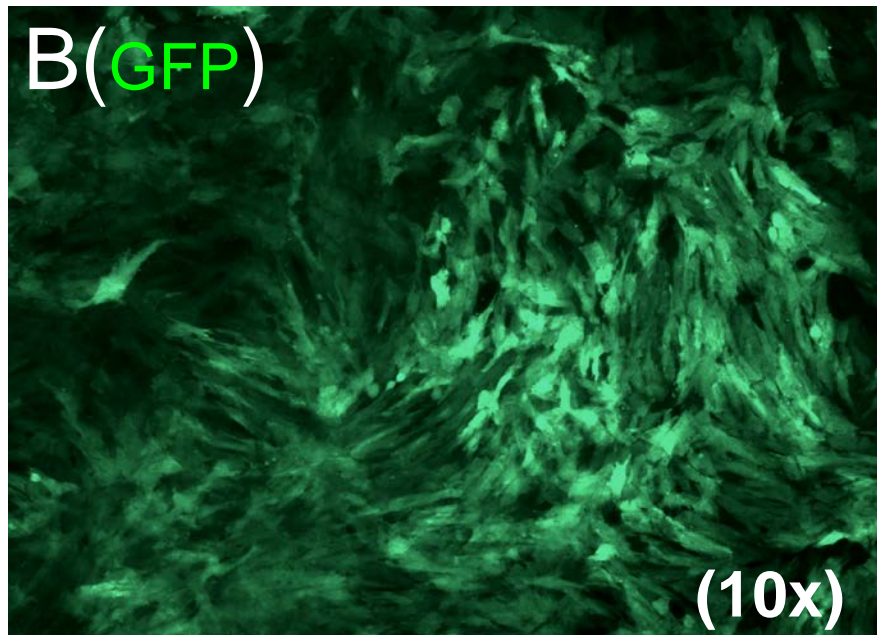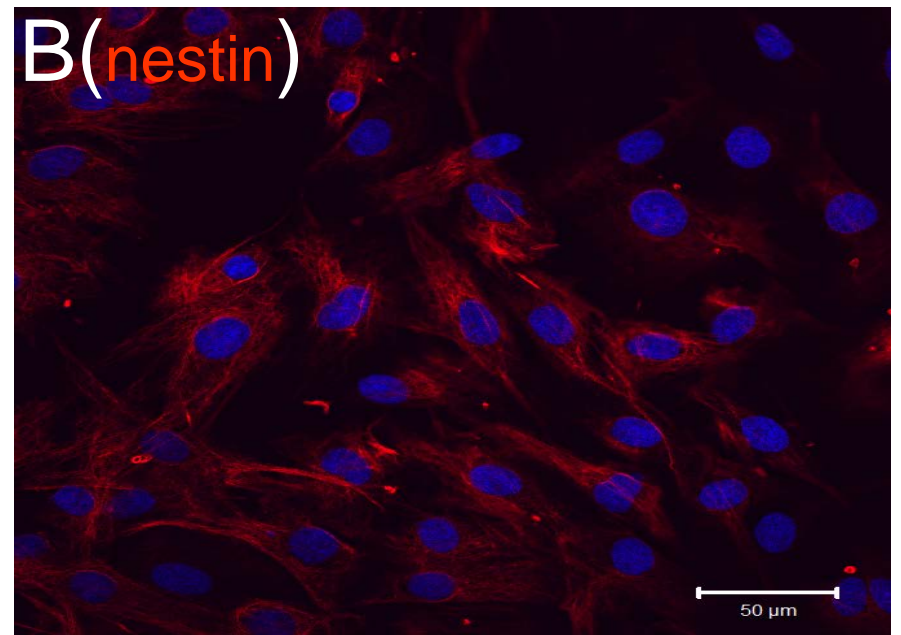

Supplement: Additional file 1: Figure S1. — Lentivirus-sHRNAmir directed against nestin. (Panel A) The infection of aortic vascular smooth muscle cells with the empty lentivirus expressing the reporter green fluorescent protein (GFP) had no significant effect on nestin immunoreactivity. (Panel B) The infection of aortic vascular smooth muscle cells with a lentivirus containing a shRNAmir directed against nestin significantly reduced staining of the intermediate filament protein. [file 12933_2014_119_MOESM1_ESM.pdf]

**Suppl Figure 2**

**SHAM CA**

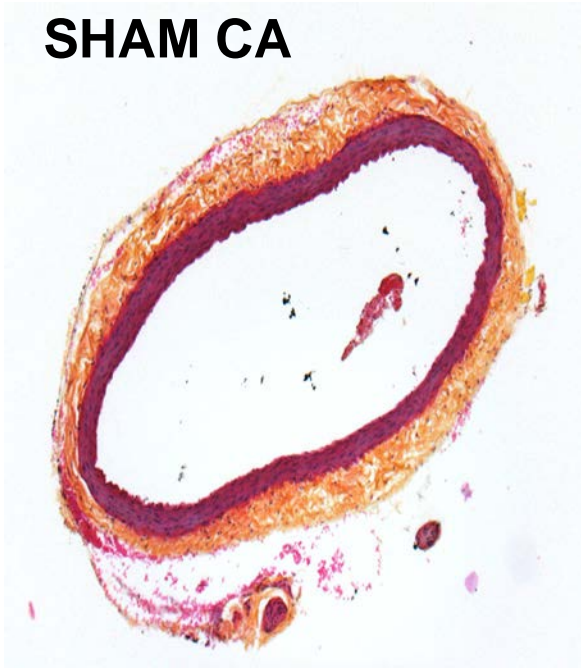

**STZ CA**

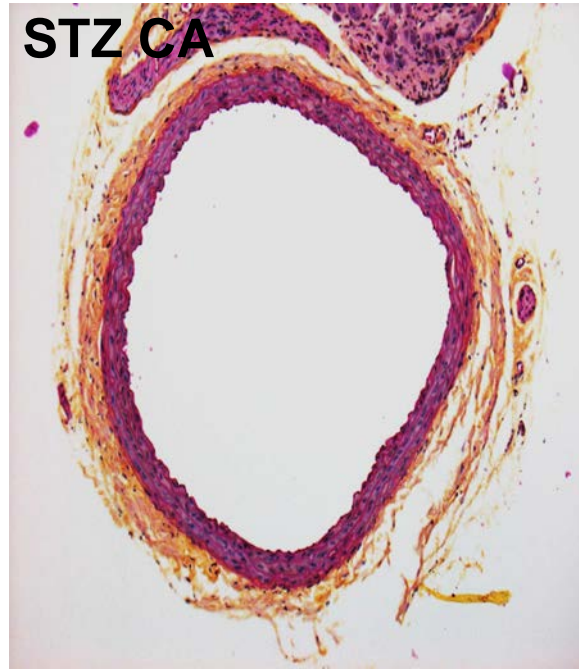

**SHAM AA**

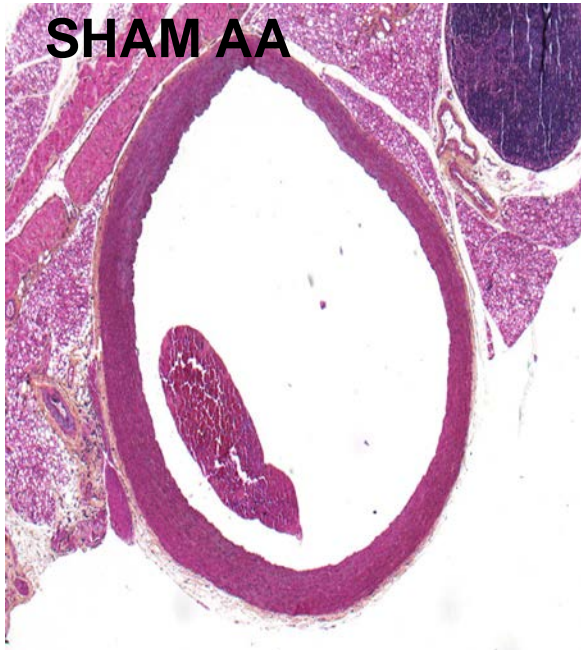

**STZ AA**

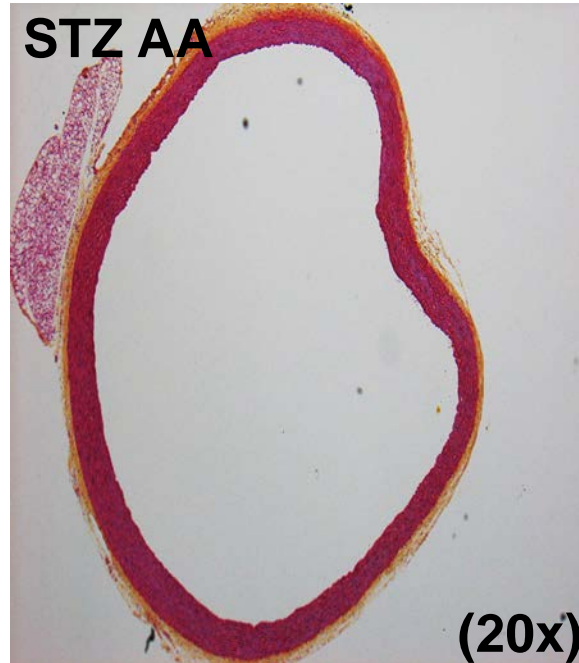

**(20x)**

Supplement: Additional file 2: Figure S2. — Vascular morphology. The media thickness and area of the carotid artery (CA) and aortic arch (AA) of streptozotocin (STZ) induced type I diabetic rats were similar to sham rats. [file 12933_2014_119_MOESM2_ESM.pdf]

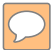

**A**

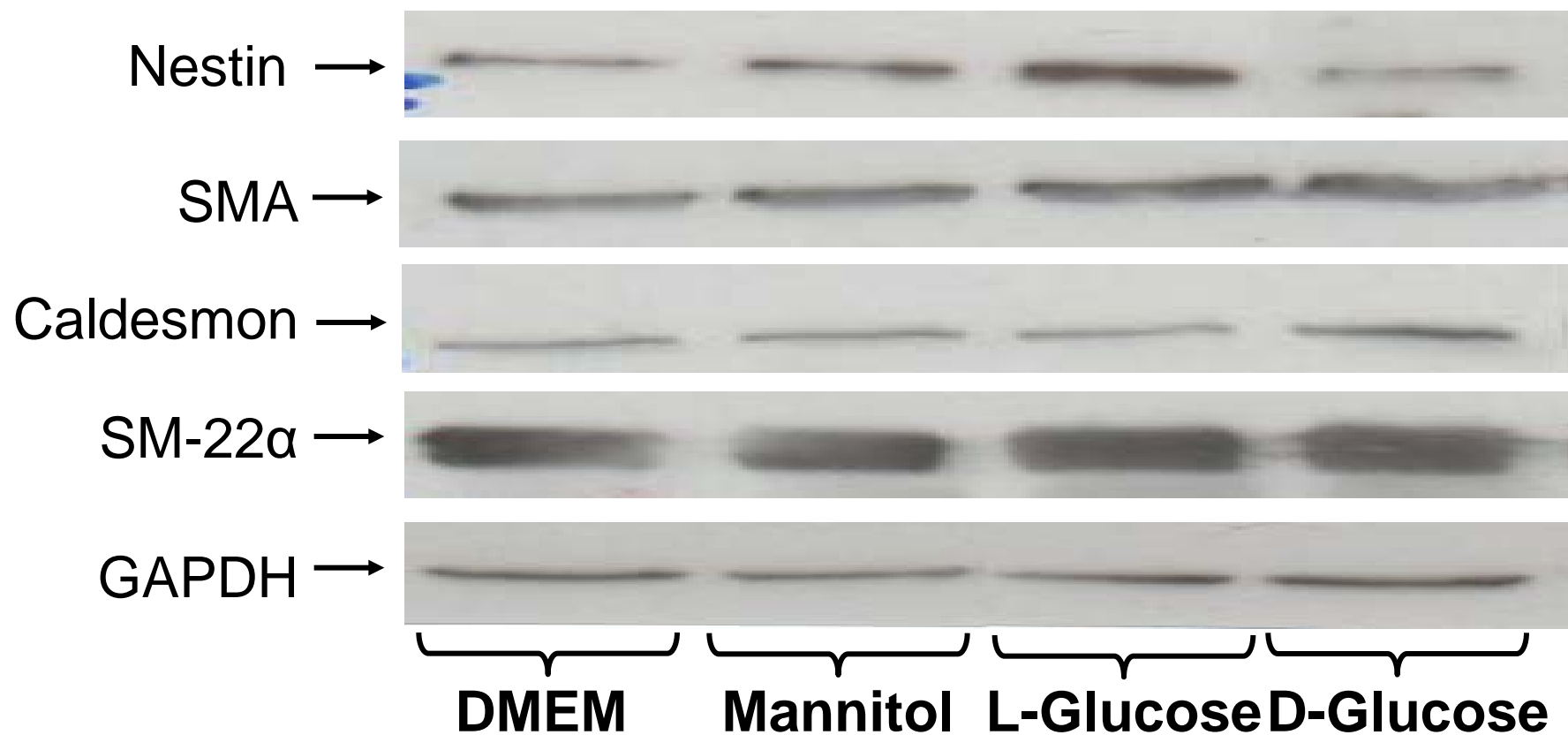

Supplement: Additional file 3: Figure S3. — Hyperglycaemia downregulated nestin protein expression in aortic VSMCs. The exposure of (Panels A & B) aortic VSMCs to 30 mM mannitol or 30 mM L-glucose for 24 hrs did not significantly influence nestin protein expression as compared to DMEM. A 48 hr exposure of aortic-derived VSMCs to 30 mM D-glucose significantly reduced nestin protein levels, as compared to 30 mM L-glucose treated cells, whereas smooth muscle α-actin (SMA), caldesmon and smooth muscle-22α protein expression was unchanged. (*) Denotes p< 0.05 versus L-glucose and data normalized to GAPDH. [file 12933_2014_119_MOESM3_ESM.zip › 3500699291311649_add7.pdf]

**B**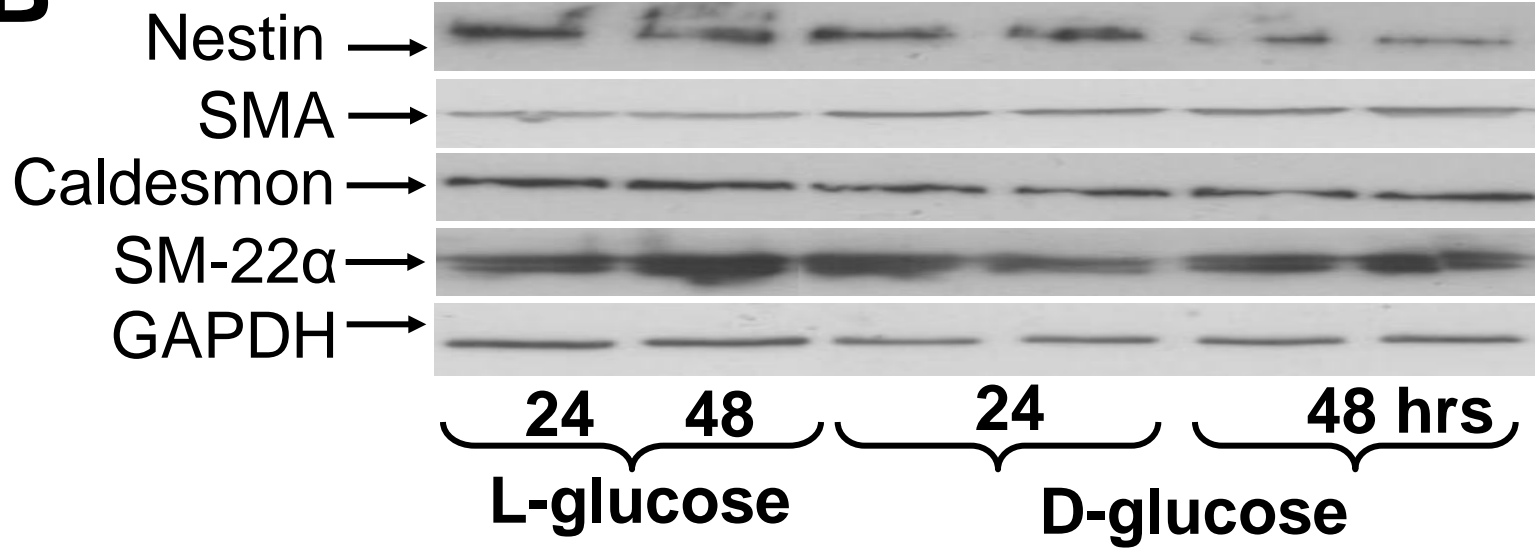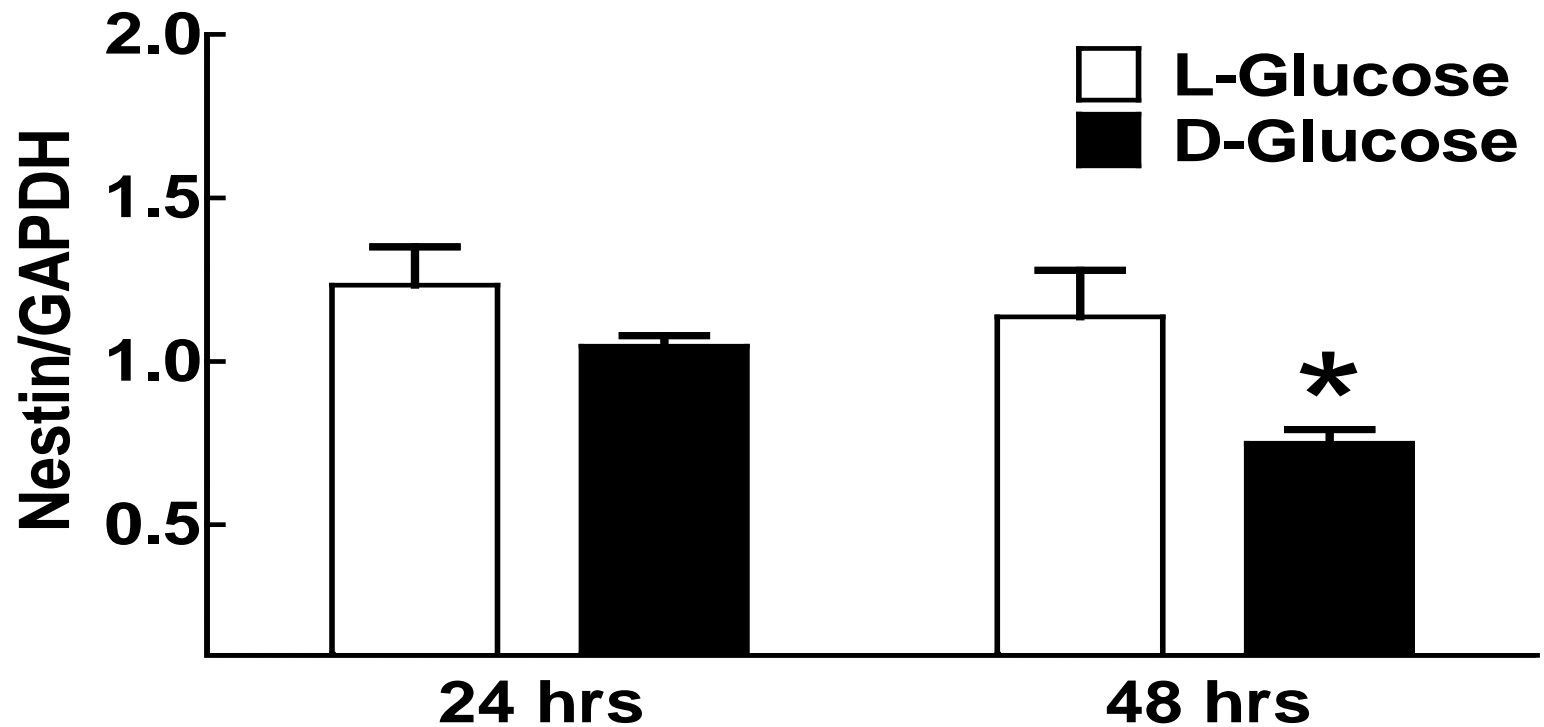

Supplement: Additional file 3: Figure S3. — Hyperglycaemia downregulated nestin protein expression in aortic VSMCs. The exposure of (Panels A & B) aortic VSMCs to 30 mM mannitol or 30 mM L-glucose for 24 hrs did not significantly influence nestin protein expression as compared to DMEM. A 48 hr exposure of aortic-derived VSMCs to 30 mM D-glucose significantly reduced nestin protein levels, as compared to 30 mM L-glucose treated cells, whereas smooth muscle α-actin (SMA), caldesmon and smooth muscle-22α protein expression was unchanged. (*) Denotes p< 0.05 versus L-glucose and data normalized to GAPDH. [file 12933_2014_119_MOESM3_ESM.zip › 3500699291311649_add8.pdf]

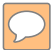**B**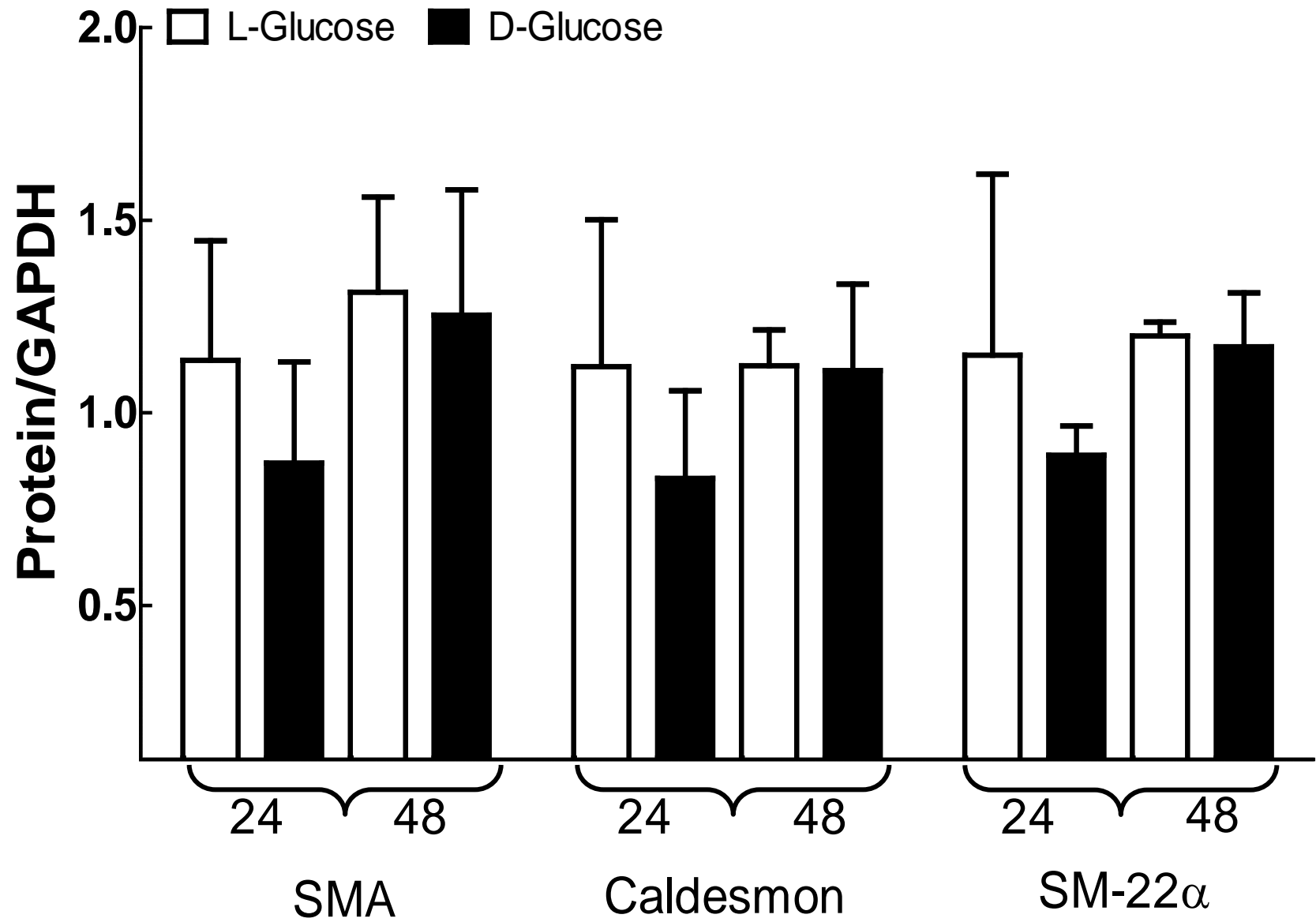

Supplement: Additional file 4: Figure S4. — The impact of hyperglycaemia on vascular smooth muscle cells. A 24 and/or 48 hour exposure of (Panel A) carotid artery (n=4-6) and (Panel B) aortic derived (n=4-6) vascular smooth muscle cells to 30 mM D-glucose had no significant effect on smooth muscle α-actin, caldesmon and smooth muscle-22α protein expression as compared to 30 mM L-glucose. Proteins levels were normalized to GAPDH. [file 12933_2014_119_MOESM4_ESM.zip › 3500699291311649_add10.pdf]

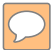**A**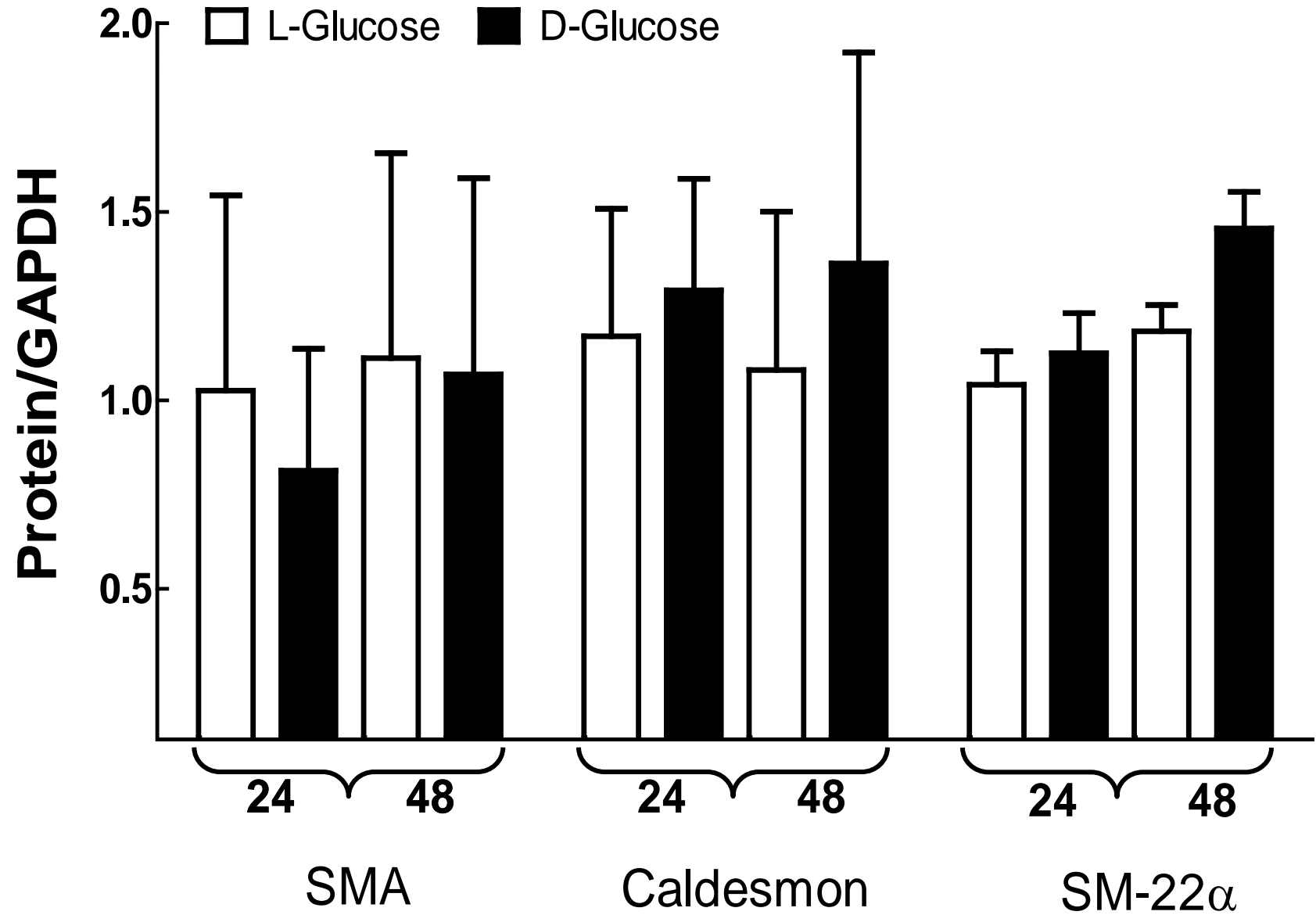

Supplement: Additional file 4: Figure S4. — The impact of hyperglycaemia on vascular smooth muscle cells. A 24 and/or 48 hour exposure of (Panel A) carotid artery (n=4-6) and (Panel B) aortic derived (n=4-6) vascular smooth muscle cells to 30 mM D-glucose had no significant effect on smooth muscle α-actin, caldesmon and smooth muscle-22α protein expression as compared to 30 mM L-glucose. Proteins levels were normalized to GAPDH. [file 12933_2014_119_MOESM4_ESM.zip › 3500699291311649_add9.pdf]
